# In-silico and in-vitro assessments of some fabaceae, rhamnaceae, apocynaceae, and anacardiaceae species against *Mycobacterium tuberculosis* H37Rv and triple-negative breast cancer cells

**DOI:** 10.1186/s12906-023-04041-5

**Published:** 2023-07-01

**Authors:** Kudakwashe Nyambo, Francis Adu-Amankwaah, Kudzanai Ian Tapfuma, Lucinda Baatjies, Lauren Julius, Liezel Smith, Mkhuseli Ngxande, Krishna Govender, Lawrence Mabasa, Afsatou Traore, Maano Valerie Masiphephethu, Idah Sithole Niang, Vuyo Mavumengwana

**Affiliations:** 1 DSI-NRF Centre of Excellence for Biomedical Tuberculosis Research, South African Medical Research Council Centre for Tuberculosis Research, Division of Molecular Biology and Human Genetics, Faculty of Medicine and Health Sciences, Stellenbosch University, Cape Town, South Africa; 2 Computer Science Division, Department of Mathematical Sciences, Faculty of Science University of Stellenbosch, Matieland, South Africa; 3 Department of Chemical Sciences, University of Johannesburg, Doornfontein Campus, P. O. Box 17011, Johannesburg, 2028 South Africa; 4 National Institute for Theoretical and Computational Sciences (NITheCS), Stellenbosch, South Africa; 5 Biomedical Research and Innovation Platform (BRIP), South African Medical Research Council (SAMRC), Tygerberg, 7505 South Africa; 6 Department of Biochemistry & Microbiology, University of Venda, Thohoyandou, South Africa; 7 Department of Biotechnology and Biochemistry, University of Zimbabwe, B064, Mount Pleasant, Harare, Zimbabwe

**Keywords:** *Mycobacterium tuberculosis*, *Schinus molle*, *Rauvolfia caffra*, LC-QTOF-MS/MS, Virtual screening, Molecular dynamics simulations, MM-GBSA, Triple-negative breast cancer, Flow cytometry, Antioxidants

## Abstract

Medicinal plants play a huge role in the treatment of various diseases in the Limpopo province (South Africa). Traditionally, concoctions used for treating tuberculosis and cancer are sometimes prepared from plant parts naturally occurring in the region, these include (but not limited to) *Schotia brachypetala, Rauvolfia caffra, Schinus molle, Ziziphus mucronate,* and *Senna petersiana. *In this study, the aim was to evaluate the potential antimycobacterial activity of the five medicinal plants against *Mycobacterium smegmatis* mc^2^155, *Mycobacterium aurum* A + , and *Mycobacterium tuberculosis* H37Rv, and cytotoxic activity against MDA-MB 231 triple-negative breast cancer cells. Phytochemical constituents present in *R. caffra* and *S. molle* were tentatively identified by LC-QTOF-MS/MS as these extracts showed antimycobacterial and cytotoxic activity. A rigorous Virtual Screening Workflow (VSW) of the tentatively identified phytocompounds was then employed to identify potential inhibitor/s of *M. tuberculosis* pantothenate kinase (PanK). Molecular dynamics simulations and post-MM-GBSA free energy calculations were used to determine the potential mode of action and selectivity of selected phytocompounds. The results showed that plant crude extracts generally exhibited poor antimycobacterial activity, except for *R. caffra* and *S. molle* which exhibited average efficacy against *M. tuberculosis* H37Rv with minimum inhibitory concentrations between 0.25–0.125 mg/mL. Only one compound with a favourable ADME profile, namely, norajmaline was returned from the VSW. Norajmaline exhibited a docking score of –7.47 kcal/mol, while, pre-MM-GBSA calculation revealed binding free energy to be –37.64 kcal/mol. All plant extracts exhibited a 50% inhibitory concentration (IC_50_) of < 30 μg/mL against MDA-MB 231 cells. Flow cytometry analysis of treated MDA-MB 231 cells showed that the dichloromethane extracts from *S. petersiana*, *Z. mucronate,* and ethyl acetate extracts from *R. caffra* and *S. molle* induced higher levels of apoptosis than cisplatin. It was concluded that norajmaline could emerge as a potential antimycobacterial lead compound. Validation of the antimycobacterial activity of norajmaline will need to be performed in vitro and in vivo before chemical modifications to enhance potency and efficacy are done. *S. petersiana*, *Z. mucronate*, *R.caffra* and *S. molle* possess strong potential as key contributors in developing new and effective treatments for triple-negative breast cancer in light of the urgent requirement for innovative therapeutic solutions.

**Supplementary Information:**

The online version contains supplementary material available at 10.1186/s12906-023-04041-5.

## Introduction

Drug resistance in breast cancer, and the escalating spread of multidrug-resistant *Mycobacterium tuberculosis* strains is a major concern because it is straining the healthcare systems, especially that of developing countries. Current tuberculosis (TB) and cancer chemotherapies in clinical use have severe side effects that often result in the development of other health-related complications. Southern Africa is one of the heavily affected regions due to a combination of various factors which include a plethora of factors usually associated with Low Income-Middle-Class Countries (LIMCC) including, inadequate health facilities, HIV-TB co-infection, and socioeconomic factors [1–3]. It is therefore critical to develop new effective communicable and noncommunicable chemotherapeutic agents that will be easily accessible to marginalized communities [4].

For most people in rural areas, the traditional pharmaceutical system is complemented by modern treatment procedures thus, broadening the scope of healthcare solutions usually available to individuals in urban areas [2, 5, 6]. Comprehensive knowledge of the diverse botanical landscape provides a baseline for prescribing complex concoctions for treating and curing various ailments. Due to strong cultural beliefs in communities embracing indigenous knowledge systems, it is not surprising to find that ethnobotanical treatment modalities are strongly adhered to [7–9]. As such, various extracts derived from plants such as *Schotia brachypetala, Senna petersiana, Ziziphus mucronata*, *Rauvolfia caffra,* and *Schinus molle,* are reported to be widely used as medicinal remedies in these communities. Traditional therapies constituting *R. caffra* have been reported as prescriptions for the treatment of ailments such as microbial infections, malaria, diabetes, diarrhea, skin infections, worm infections, and coughs [10, 11]. Oils extracted from *S. molle* have been associated with nutritional, antimicrobial, anti-inflammatory, anti-depressant, astringent, stimulant, and anti-cancer activity [12, 13]. Extracts from the bark of *Z. mucronata* were shown to exhibit broad-spectrum antimicrobial activity [14]. Ethnobotanical knowledge can be used to harness the prominent plant arsenal by selecting the species of plants that are prescribed as antibacterial remedies.

With the increase of drug-resistant *M. tuberculosis* strains, the adoption of *in-silico* techniques enables efficient and cost-effective identification of potential lead compounds that can further be developed into potent drugs [15]. Targeting *M. tuberculosis* enzymes that participate in essential biosynthetic pathways with bioactive phytocompounds may lead to the discovery of novel scaffolds with novel mechanisms of action. *M. tuberculosis* pantothenate kinase (PanK) is a critical regulatory target that catalyzes the first and rate-limiting step of the biosynthesis of the CoA pathway. CoA is a crucial cofactor for the survival of the bacilli because it is vital for enzymes involved in lipid biosynthesis and catabolism. Lipids are essential building blocks for the cell envelope and serve as *M. tuberculosis* virulence factors [16, 17]. From this perspective, a targeted *in-silico* exploration of the ethnopharmacological derived compounds present in crude extracts against *M. tuberculosis* PanK may identify promising lead scaffolds. Herein, the study aims to evaluate the antimycobacterial activity of the crude extracts of *S. brachypetala, S. petersiana, Z. mucronata*, *R. caffra,* and *S. molle* and provide a detailed insight into compounds that could have complimentary conformational features required for binding in the PanK domain. Virtual screening workflow, molecular dynamics (MD) simulations, and MM-GBSA binding free energy were performed to reveal a new dimension on the dynamics of targeting the PanK with plant-derived ligands.

Breast cancer, particularly the aggressive triple-negative breast cancer subtype, remains a major worldwide health concern [18]. It is critical to develop innovative treatment techniques against this subtype. The plant species selected in this study have an abundance of structurally diverse secondary metabolites which include indole alkaloids, phenols, terpenoids, and flavonoids. These secondary metabolites are well known for possessing anticancer activity [19, 20], thus, the second aim of the study was to explore the antiproliferative activity of the crude extracts of *S. brachypetala, S. petersiana, Z. mucronata*, *R. caffra,* and *S. molle* against MDA-MB 231, a triple-negative breast cancer cell line. Understanding their antiproliferative effects could pave the way for the development of new therapeutic interventions for breast cancer.

## Materials and method

### Plant collection and preparation

In this study, medicinal plants (supplementary material, appendix: Table A1) were collected in Tshififi, Siambe, and Lufule villages, Vhembe district, Limpopo province, South Africa. Voucher specimens were identified and authenticated by Professor P. Tshisikhawe at the UNIVEN herbarium (Department of Botany, University of Venda). Plant samples were separated into leaves, bark, and roots and the different parts were then dried at ambient temperature in the laboratory for two weeks. Thereafter, they were separately ground into a fine powder and kept in airtight containers in the dark until use. The crude ingredients present in all the plant material were exhaustively extracted by dissolving 10 g of each plant material in 100 mL of hexane, chloroform, dichloromethane, ethyl acetate, acetone, ethanol, and methanol (Merck, Kenilworth, NJ, USA). The solutions were shaken for one hour at 200 rpm, then the supernatant was filtered into pre-weighed bottles. The process was repeated three times to exhaustively extract phytocompounds from the plant residues. Thereafter, crude plant extracts from respective solvents were pooled and concentrated by drying under a constant stream of ambient air in the fume hood. Dry extracts were then stored at 4 °C until further analysis.

### Antimycobacterial minimum inhibitory concentration assay

The antimycobacterial activity was performed to evaluate the activity of the crude extracts against *Mycobacterium smegmatis* mc^2^155, *Mycobacterium aurum* A + , and *M. tuberculosis* H37Rv as described [21]. Briefly, the Mycobacteria were cultured in Middlebrook 7H9 (Fluka M7H9) broth supplemented with 0.2% glycerol, 0.05% Tween 80, and 10% Middlebrook growth supplement OADC (oleic acid, albumin, dextrose, and catalase) at 37 °C. The minimum inhibitory concentration (MIC) to obstruct *M. smegmatis*, *M. aurum*, and *M. tuberculosis* growth were determined following a previously described method with modest changes [21]. Dried plant extracts were dissolved in dimethyl sulfoxide (DMSO) to a final concentration of 4 mg/mL which was followed by a twofold serial dilution in 96-well microtiter plates to achieve a series of concentrations ranging from 0.2–2.0 mg/mL. DMSO was used as a negative control, while isoniazid was used as a positive control. *M. smegmatis* and *M. aurum* plates were incubated at 37 °C for 72 h, while *M. tuberculosis* plates were incubated for seven days before adding 20 µL of 0.02% resazurin. The non-pathogenic strains were incubated for a further four hours and *M. tuberculosis* for a further 24 h. Growth inhibition was indicated by a constant blue resazurin colour, while a pink colour indicated the inactivity of extracts against Mycobacteria. All extracts were tested in triplicate.

### Tentative identification of phytochemicals

A liquid chromatography system connected to quadrupole time-of-flight with tandem mass spectrometry (LC-QTOF-MS/MS) was employed to identify the plant crude extracts as previously described [21]. The system consists of a Waters Acquity ultra-performance liquid chromatography (U-PLC) with an Acquity photo-diode array (PDA) detector, coupled to a Waters Synapt G2 quadrupole time-of-flight mass spectrometer (Milford, MA, USA). The plant metabolites were chromatographically separated using a Waters UPLC BEH C18 column (1.7 µm particle size, 2.1 $\times$ 100 mm, Waters Corp). Deionized water acidified with 0.1% formic acid (v/v) was used as solvent A, while acetonitrile was used as solvent B were in a gradient elution program set as follows: 0% solvent B between 0–0.5 min; 0–100% solvent B between 0.5–12.00 min; 100% solvent B between 12.00–12.50 min; 100–0% solvent B between 12.50–13.00 min; 0% solvent B between 13.00–15.00 min [21]. The spectral data were acquired at 150 to 1500 m*/z* in positive centroid mode. Ionization was achieved with an electrospray source using a cone voltage of 15 V and capillary voltage of 2.5 kV. Nitrogen was used as the desolvation gas at 650 L/hr and the desolvation temperature was set to 275 °C. The raw data containing spectral data were converted to.abf format. The.abf files were then processed using the MS-Dial module (version 4.24) and MS-Finder (version 3.5) for tentative identification of compounds using the following parameters: error ppm < 7.0, [M + H]^+^ adducts ions. Manual annotation of compounds was also performed using KNapSacK (http://www.knapsackfamily.com/KNApSAcK_Family/) and Metfrag (https://msbi.ipb-halle.de/MetFrag/) compound databases.

### Virtual screening of tentatively identified compounds

#### Receptor preparation

The raw crystal structure of *M. tuberculosis* pantothenate kinase (PanK) PDB:4BFX was downloaded from the protein data bank (https://www.rcsb.org/structure/4BFX). The structure was prepared as described [22] in Schrödinger (Release 2021–1) using the Protein Preparation Wizard module. Briefly, hydrogen atoms were added, the loop region was refined, H-bond assignments were optimized, and energy was minimized by an OPLS-4 force field. The coordinates of the co-crystallized ligand (1f) [23] were used for the generation of the binding domain using the Receptor Grid Generating module (Schrödinger Release 2021–1).

#### Ligand preparation

The compounds were prepared as previously described [21]. Briefly, the LigPrep module [24] (Schrödinger Release 2021–1) was used to prepare the compounds following these parameters: energy minimized by an OPLS-4 force field, generate ionization states at pH 7.0 + 2.0 and generate at most 32 conformers per each ligand to develop a new set of 640 possible compounds. The prepared library was subjected to a molecular docking-based virtual screening.

#### Structure-based virtual screening of compounds

A Virtual Screening Workflow (VSW) comprised of the following modules (Schrödinger Release 2021–1) [25]: QickProp, Lipinski’s Rule of five filters, high-throughput virtual screening (HTVS), standard precision (SP), and lastly, extra-precision, which were used for screening the library of phytocompounds to obtain a hit list. QickProp module filtered the phytocompounds based on features of ADME (absorption, distribution, metabolism, and excretion). The obtained compound list was further subjected to Lipinski’s rule of five filters. Using the Glide module, the returned compounds were subjected to three-step docking regimes with increasing precision. Briefly, the compounds were docked against PanK using high-throughput virtual screening (HTVS), standard precision (SP), and lastly extra-precision (XP). Only 20% of the HTVS docking hits were applied to SP docking. Only 20% of SP docking outputs were subjected to XP docking, from which 30% were retrieved as described [26]. The pre-MM-GBSA (Molecular Mechanics, the Generalized Born model, and Solvent Accessibility) was performed to evaluate the free binding energy (ΔG_bind_) of the protein–ligand complex/es as described [25].

#### Molecular dynamics simulation

The selected poses for the PanK-phytocompound complex, PanK-control ligand (1f) complex, and native unbound PanK conformations were subjected to molecular dynamics (MD) simulations using Desmond (Schrödinger Release 2021–1) to evaluate the stability of the docked complex. A total of three separate MD systems were created according to the same parameters as described [21]. Briefly, the protein–ligand complex was explicitly solvated by enclosing it in an orthorhombic TIP3P water box with the protein surface atoms 10 Å away from the box boundary. The system was neutralized by adding 0.15 M counter ions (Na^+^ and Cl^–^). All systems had implemented periodic grid conditions, long-range electrostatic interactions were generated for the particle-mesh Ewald method with a non-bonding cut-off distance of 12 Å. The systems were energy minimized and equilibrated at constant pressure and temperature (1.01325 bar and 303.15 K, respectively) with Nose–Hoover thermostat, and Martyna-Tobias-Klein as the default barostat with a 2.0 ps interval by applying an isotropic coupling style. The internal energy was stored for every 1000 ps of the actual frame. The NPT ensemble MD simulations were performed for a duration of 50 ns. The stability for each complex was evaluated by computing the root-mean-square deviation (RMSD), and root-mean-square fluctuations (RMSF). The molecular mechanics generalized Born surface area (MM-GBSA) (ΔG_bind_) (kcal/mol) binding free energies were computed based on Molecular Mechanics + Implicit Solvent Energy Function [27].

### Determination of cytotoxic effects of plant crude extracts

The cytotoxic effect of plants against triple-negative breast cancer cells (MDA-MB 231) was conducted as previously described with minor modifications [28]. MDA-MB 231 cells (passage number 43) were donated by Prof Anna-Mart Engelbrecht, Stellenbosch University, South Africa. Briefly, cells were seeded in a 96-well plate at a density of 6000 cells/well and left to attach for 24 h. The plant crude extracts were dissolved in dimethyl sulfoxide (DMSO) to form a 100 mg/mL stock solution. MDA-MB 231 cells were treated with plant crude extracts (62.5–250 μg/mL) and cisplatin (reference drug at 3 μg/mL, Sigma Aldrich, USA) for 48 h. Spent Dulbecco's modified Eagle's medium (DMEM) (Sigma Aldrich, USA), supplemented with 10% foetal bovine serum (DMEM complete medium) was removed and replaced with 0.5 mg/mL 3-(4,5-Dimethylthiazol-2-yl)-2,5-diphenyltetrazolium bromide (MTT) (Sigma Aldrich, USA) dissolved in DMEM complete medium. After four hours of incubation, MTT solution was removed, and the formazan product dissolved in 100 μL DMSO. Absorbance was measured at 540 nm using a microtiter plate reader (FLUOstar Omega, BMG Labtech, Germany) [28]. All incubations were done in a humidified incubator (ESCO, Vivid Air) with 5% CO_2_ at 37 °C.

### Cytotoxic effects of plant crude extracts against HepG2/C3A and vero cell lines

The cytotoxic effect of HepG2/C3A and Vero cells were studied as previously described [29, 30]. In brief, Hep G2 clonal derivatives (C3A) (human hepatoma cells) with passage number 14 and Vero cells (normal monkey kidney epithelial cells) with passage number 11 were used to evaluate the cytotoxicity of plant extracts. These cells were purchased from Cellonex, South Africa, by Prof Maryna van de Venter, Nelson Mandela University South Africa. Cells were cultured at 37 °C in a humidified incubator with 5% CO_2_ in 10 cm culture dishes.

The complete growth medium consisting of Eagle's minimal essential medium (EMEM) supplemented with 10% FBS, 1% penicillin–streptomycin (penstrep), and 1 × Non-Essential Amino Acid (NEAA) (GE Healthcare Life Science, Logan, UT, USA), was used to grow the HepG2 cells, while DMEM supplemented with 10% FBS and 1% penstrep was used to grow Vero cells. Both cell lines were respectively seeded in 96-well microtiter plates at a density of 4 000 cells per well using their respective media and incubated overnight at 37 °C, 5% CO_2_, and 100% relative humidity to allow for cell attachment. Thereafter, cells were treated with 100 µL aliquots of extracts at 50, 100, and 200 μg/mL, and 10, 20, and 40 μM melphalan (positive control). Incubation was then performed for a further 48 h. After incubation, the treatment media was aspirated from all the wells and 100 μL of Hoechst 33342 nuclear dye (5 μg/mL) was added to each well and incubated for 20 min at room temperature. Cells were stained with propidium iodide (PI) at 100 μg/mL to enumerate the proportion of dead cells within the population. Cells were imaged immediately after adding PI, using the ImageXpress Micro XLS Widefield Microscope (Molecular Devices) with a 10 × Plan Fluor objective and DAPI and Texas Red filter cubes [29, 30].

### Annexin V-FITC/PI apoptosis assay

MDA-MB 231 cells were seeded at a density of 2.5 × 10^5^ cells/well in 24-well plates and incubated overnight at 37 °C in a humidified incubator with 5% CO_2_ (ESCO, Vivid Air). Cisplatin (10 μM/3 μg/mL) and plant crude extracts at their respective IC_50_ values were used to treat the cells for 48 h. Following the incubation period, the cells were detached by adding 80 μL of Accutase™ for 10 min or until cells were detached. One milliliter complete media were added to each well and incubated for an hour to allow cells to recover. The cells transferred to polypropylene flow cytometry tubes and harvested by centrifugation (1500 rpm) for 5 min at 4 °C. The pellets were washed with ice-cold DMEM complete media and centrifuged (1500 rpm) for 5 min at 4 °C. The Annexin V FITC/PI apoptosis detection kit was used to stain the cells as per manufacturer’s instructions (Invitrogen, Thermo Fisher Scientific). The pellets were redissolved in ice-cold 1 × binding buffer. To each tube, 1 μL of Annexin V FITC and 5 μL of PI were added. Control tubes with single stains were also added and incubated in the dark for 15 min. After incubation, 400 μL of 1 $\times$ annexin-binding buffer was added and gently mixed. The samples were read on a BC DxFlex flow cytometer (Beckman Coulter, USA) [28].

### Statistical analysis

The statistical analysis of the behavioural data was conducted using the student t-test with GraphPad Prism (GraphPad Software Inc., San Diego, CA) and Microsoft Excel. The mean values ± standard deviation (SD) were reported for all data. Statistical significance was determined at a significance level of *p* ≤ 0.05, indicating that differences with this level of probability or lower were considered statistically significant.

## Results

### Antimycobacterial activity

Crude extracts were extracted using seven different solvents of varying polarity. A total of 30 extracts obtained from *S. brachypetala, R. caffra, S. molle, Z. mucronata,* and *S. petersiana* were evaluated for antimycobacterial activity against *M. smegmatis* mc^2^155, *M. aurum* A + *,* and *M. tuberculosis* H37Rv. The MIC value of > 2 mg/mL was selected as a cutoff for all Mycobacterial strains’ susceptibility. The crude exhibited varying antimycobacterial activity (Tables 1, 2, 3). All the extracts exhibited poor inhibition against *M. smegmatis* mc^2^155. In contrast, the control (isoniazid) showed an MIC of 0.03 mg/mL against *M. smegmatis* mc^2^155 (Table 1).

**Table 1:** Minimum inhibitory concentration (MIC) of crude plant extracts against *M. smegmatis* mc^2^155

Plant species	Extracts (mg/mL)							
	**Hexane**	**Chloro-form**	**Dichloro-methane**	**Ethyl acetate**	**Acetone**	**Ethanol**	**Methanol**	**INH**
*S. brachypetala*	> 2	> 2	> 2	> 2	> 2	> 2	> 2	–
*R. caffra*	> 2	> 2	> 2	> 2	> 2	> 2	> 2	–
*S. molle*	> 2	> 2	> 2	> 2	> 2	> 2	> 2	–
*Z. mucronata*	> 2	> 2	> 2	> 2	> 2	> 2	> 2	–
*S. petersiana*	> 2	> 2	> 2	> 2	> 2	> 2	> 2	–
^**a**^**Control**	–	–	–	–	–	–	–	0.03

^a^ INH (isoniazid) was used as a positive control

**Table 2:** Minimum inhibitory concentration (MIC) of crude plant extracts against *M. aurum* A +

Plant species	Extracts (mg/mL)							
	**Hexane**	**Chloro-form**	**Dichloro-methane**	**Ethyl acetate**	**Acetone**	**Ethanol**	**Methanol**	**INH**
*S. brachypetala*	> 2	2	> 2	> 2	> 2	> 2	> 2	–
*R. caffra*	0.13	0.04	0.07	0.07	0.13	0.5	0.25	–
*S. molle*	0.04	0.02	0.25	0.02	0.04	> 2	0.04	–
*Z. mucronata*	2	2	0.04	> 2	2	> 2	0.25	–
*S. petersiana*	> 2	2	0.04	> 2	> 2	> 2	> 2	–
^a^Control	–	–	–	–	–	–	–	0.03

^a^ INH (isoniazid) was used as a control

**Table 3:** Minimum inhibitory concentration (MIC) of crude plant extracts against *M. tuberculosis* H37Rv

**Plant species**			**Extracts (mg/mL)**						
	**Hexane**	**Chloroform**		**Dichloro-methane**	**Ethyl acetate**	**Acetone**	**Ethanol**	**Methanol**	**INH**
*S. brachypetala*	> 2	> 2		> 2	> 2	> 2	> 2	> 2	–
*R. caffra*	> 2	> 2		0.25	> 2	> 2	> 2	> 2	–
*S. molle*	> 2	> 2		0.125	> 2	> 2	> 2	> 2	–
*Z. mucronata*	> 2	> 2		> 2	> 2	> 2	> 2	> 2	–
*S. petersiana*	> 2	> 2		> 2	> 2	> 2	> 2	> 2	–
^a^Control	–	–		–	–	–	–	–	< 0.31

^**a**^ INH (isoniazid) was used as a control

All the crude extracts from *R. caffra* showed strong efficacy against *M. aurum* A + (Table 2). While, hexane, chloroform, dichloromethane, ethyl acetate, acetone, and methanol extracts of *S. molle* strongly exhibited *M. aurum* A + , dichloromethane and methanol crude extracts from *Z. mucronata* showed potent efficacy against *M. aurum* A + . While for *S. petersiana* only the dichloromethane extract strongly inhibited *M. aurum* A + , *S. brachypetala* exhibited poor inhibitory activity against *M. aurum* A + .

Crude extracts from *R. caffra* and *S. molle* were observed to possess antimycobacterial activity for *M. tuberculosis* (Table 3). *S. molle* exhibited higher anti-*M. tuberculosis* activity (MIC = 0.125 mg/mL) than *R. caffra* (MIC = 0.25 mg/mL). *R. caffra* and *S. molle* crude extracts may possess useful bioactive constituents that may have the potential to serve as drug leads. Plants are undoubtedly an invaluable bio-factory comprised of numerous diverse bio-active ingredients. Therefore, the constituents present in crude extracts of *R. caffra* and *S. molle* were tentatively identified by untargeted LC-QTOF-MS/MS.

### Tentatively identification of phytocompounds

The active phytoconstituents present in the *R. caffra* and *S. molle* crude extracts were tentatively identified using LC–MS-QTOF. Basically, the tentatively identified phytocompounds exhibited varied mass to charge ratio (*m/z*) values ranging from 117.1031 to 513.2248. The tentatively identified compounds are represented in Table 4. The classes of constituents present in *R. caffra* were mostly alkaloids, terpenoids, indole alkaloids, and glycoalkaloids (Table 4), while for *S. molle* the compounds present were mostly terpenoids, terpenes, sesquiterpenes, and triterpenoid saponins.

**Table 4:** Tentatively identified compounds present in *R. caffra* crude extract

RT (min)	Peak height	Precursor *m/z*	Molecular formula	Error ppm	Compound	Class
2.8152	1644.42	171.1031	C_9_H_14_O_3_	8.9362	Boonein	Terpenoid
3.4693	7986.07	313.1922	C_19_H_24_N_2_O_2_	3.6591	Norajmaline	Indole Alkaloid
3.9888	8118.74	513.2248	C_27_H_32_N_2_O_8_	3.2305	Raucaffricine	Glucoalkaloid
4.4711	12250.83	351.1713	C_21_H_22_N_2_O_3_	2.7935	Raucaffrine	Alkaloid
4.0898	5624.79	327.2076	C_20_H_26_N_2_O_2_	2.7352	Ajmaline	Alkaloid
4.2318	2396.07	355.2032	C_21_H_26_N_2_O_3_	4.4509	Acetylnorajmaline	Alkaloid
4.3590	2533.79	367.1664	C_21_H_22_N_2_O_4_	3.1756	Apodine	Alkaloid
4.7589	3064.10	339.1703	C_20_H_22_N_2_O_3_	–0.056	Akuammicine N-oxide	Alkaloid
4.8187	13412.6	353.1863	C_21_H_24_N_2_O_3_	0.9371	Raucaffrinoline	Indole Alkaloid
4.8710	1292.01	323.1758	C_20_H_22_N_2_O_2_	1.2253	Norpurpeline	Indole Alkaloid
5.0131	2402.98	349.1563	C_21_H_20_N_2_O_3_	4.6712	Alstonine	Indole Alkaloid
5.4280	2063.06	383.1618	C_21_H_22_N_2_O_5_	4.3115	Apodinine	Alkaloid
7.1101	1719.26	357.1816	C_20_H_24_N_2_O_4_	2.0045	Compactinervinete	Alkaloid

### *In-silico* screening of the tentatively identified compounds

The library of the tentatively identified compounds was screened by a Virtual Screening Workflow (VSW) (Qikprop, Lipinski’s rule of 5, HVTS, SP, and XP docking) to filter and reduce false positive hit compounds (Table 5). The XP docking was performed to precisely search for the best protein–ligand complementarity conformation. Norajmaline was returned as a potential hit from the extensive filtering stages and exhibited an XP docking score of –7.465 kcal/mol (Table 5). The best-hit compound, norajmaline, returned from the rigorous VSW, exhibited zero violations for Lipinski’s rule of five, the percentage human oral absorption was 63.99%, Van der Waals surface area of polar nitrogen and oxygen atoms (PSA) was 61.53, QPlogS was –0.81, Solvent accessible surface area (SASA) was 532.97, and the dipole value was 1.59. Overall, the ADME values were promising as they were all in the recommended ranges. The pre-MD simulation binding energy (ΔG_bind_) of norajmaline-PanK was –37.64 kcal/mol.

**Table 5:** The predicted ADME features (SASA, dipole, Qplogs, % Human Oral Absorption and PSA), and the molecular docking XP score and Pre-MM-GBSA (ΔG_bind_) values of norajmaline against 4BFX

Compound ID	mol MW (170–725)	Dipole (1.0–12.5)	SASA	^a^QplogS (− 6.5 to 0.5)	PSA (7.0–200.0)	Volume	%Human Oral Absorption	Rule of Five	XP GScore (kcal/mol)	ΔG_bind_ (kcal/mol)
Norajmaline	318.46	1.59	532.97	**–**0.81	61.53	991.26	63.99	0	**–**7.47	**–**37.64

^a^QPlogS-(Predicted aqueous solubility, log S. S in mol dm^–3^ is the concentration of the solute in a saturated solution that is in equilibrium with the crystalline solid.)

**Fig. 1:**
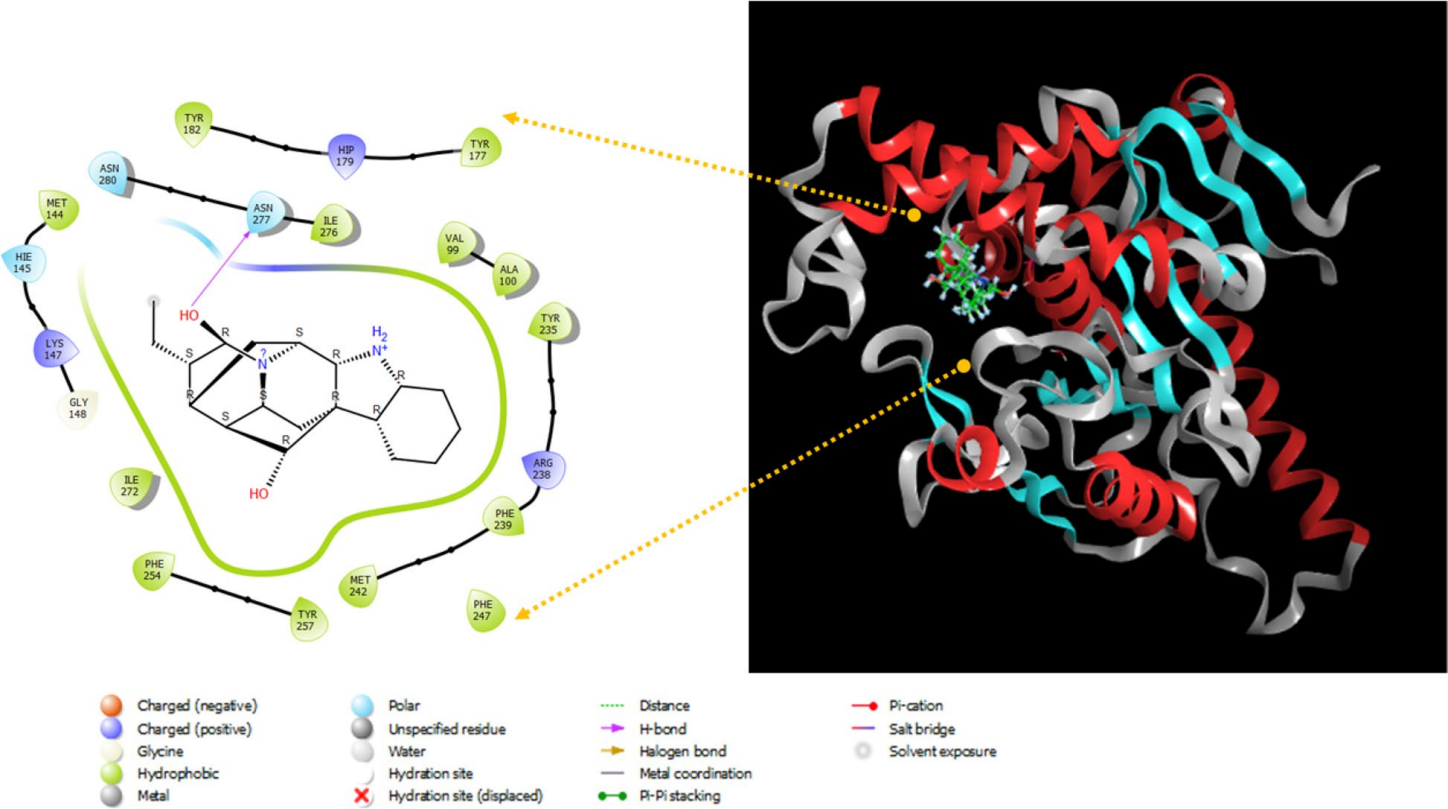
Docked orientation and interaction of norajmaline with PanK residues in the binding site

Analyses of the best XP docked configuration depicted in Fig. 1, revealed that norajmaline is buried in the hydrophobic internal cavity of the protein. The main driving forces involved in the binding of norajmaline against amino acid residues of PanK were predicted to be predominantly hydrophobic interactions (Try257, Met242, Phe239, Tyr235, Ala100, Val99, Ile276, Met144, Ile272, Phe254, Try177, and Tyr1820). In contrast, ASN277 was involved in hydrogen bonding, while (Arg238, His179, and Lys147) were involved in positively charged interactions and polar interactions (Asn280, Asn277, and Hie145). The PanK-norajmaline complex, unbound PanK, and PanK-control ligand (1f) complex were further subjected to molecular dynamics simulations and free-binding energy calculation for the complex.

**Fig. 2:**
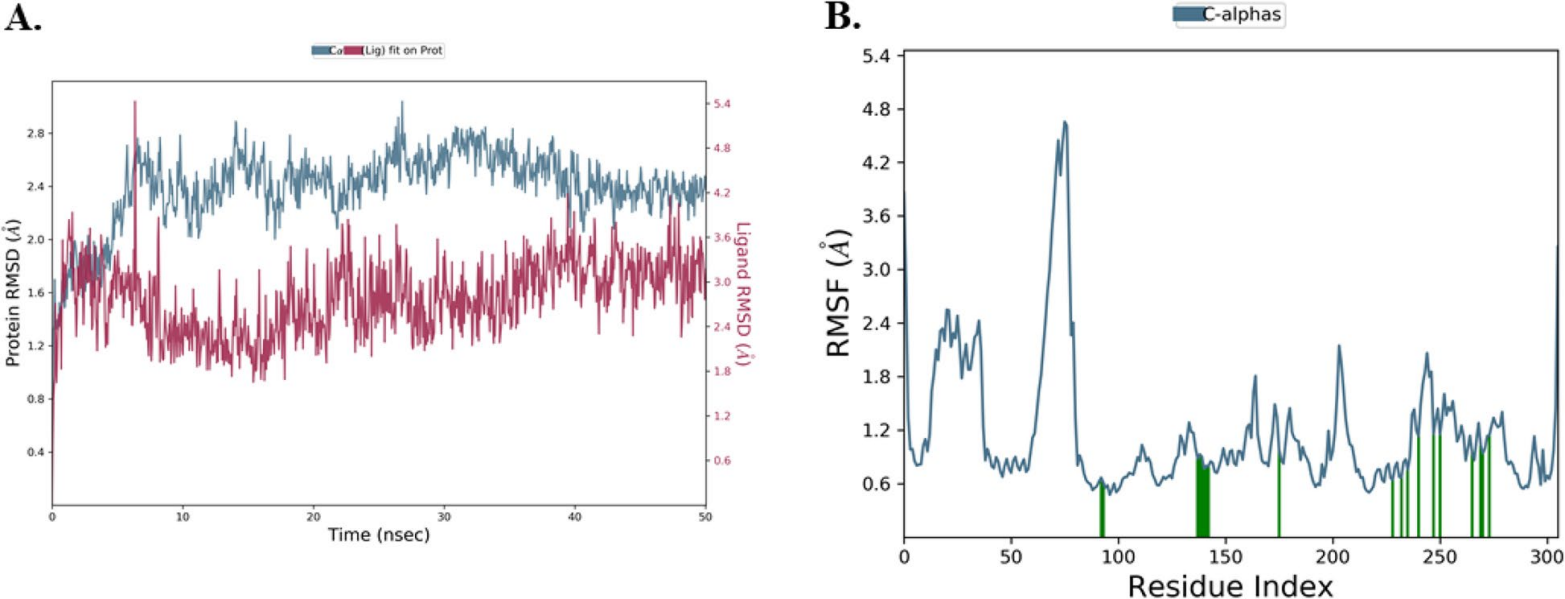
Molecular dynamics simulation of PanK complexed with norajmaline. In the figure, **A** shows the RSMD of C-α-residues of PanK observed during a 50 ns simulation. **B** shows the RMSF of C-α-residues of PanK, where the green lines indicate the residues of 4BFX in contact with the ligand during the simulation

MD simulations were performed to provide a comprehensive insight into the structural dynamics of the binding of norajmaline in the hydrophobic cavity of the PanK. Root Mean Square Deviation (RMSD) of the PanK-norajmaline complex and the unbound cα atoms were performed to evaluate the stability of the protein–ligand complex. The RMSD profile of bound PanK Cα-atoms shows a steep increase in deviation from 1.6 Å at 0 ns to approximately 2.6 Å at 8 ns, was then maintained between 2.4 Å and 2.6 Å up to 30 ns and then gradually decreased to 2.3 Å up to 50 ns as depicted in Fig. 1. The sharp increase observed from 0 to 8 ns indicates a change in PanK confirmation as it interacts with the norajmaline (Fig. 2A). The Norajmaline RMSD (Fig. 1) was maintained at approximately 3.0 Å during the 50 ns simulation, which illustrates the ligand was fairly undergoing slight conformational changes. The RMSF of PanK illustrates a large fluctuation at residues between 0 and 100, while smaller fluctuations were observed from residues 100–300 which were participating in interacting with the ligand (Fig. 2B). According to the RMSD, the protein–ligand complex was observed to be stable during the 50 ns simulation (Fig. 2A).

**Fig. 3:**
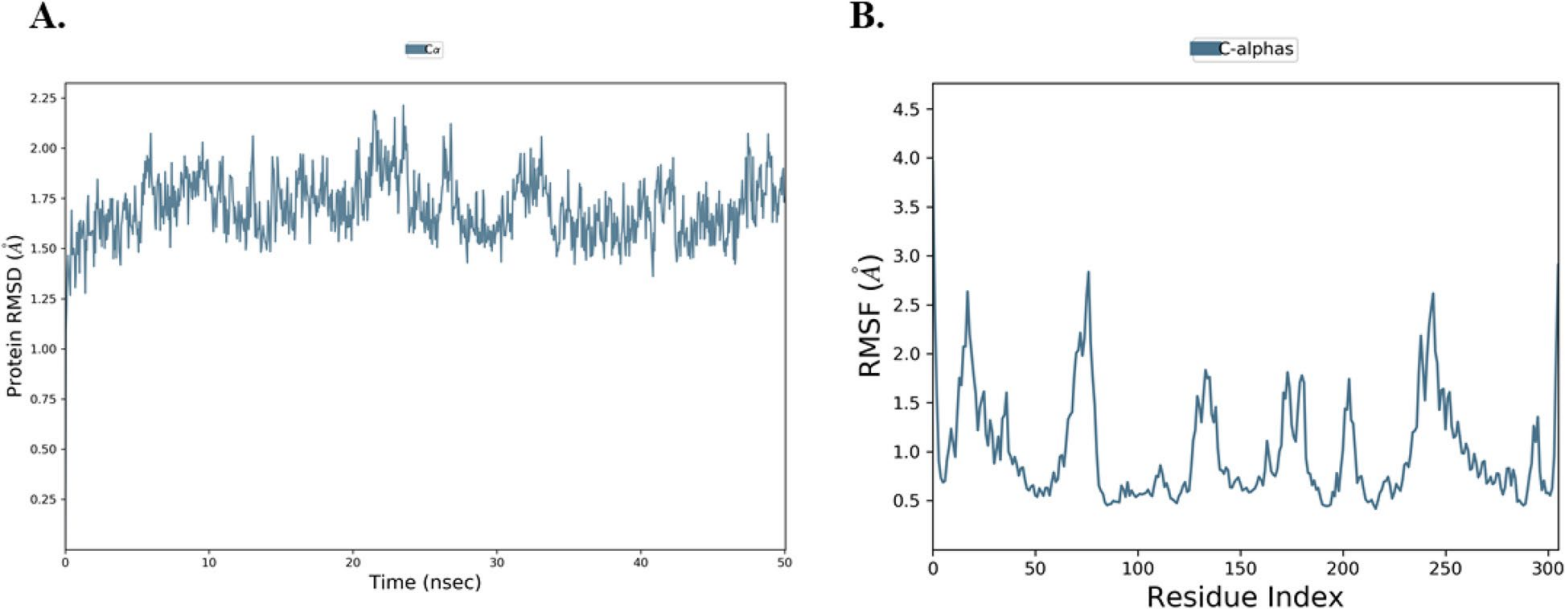
Simulated native unbound PanK. In **A**, the RMSD of PanK C-α-residues observed during a 50 ns simulation is shown. In **B** the RMSF of PanK C-α-residues is shown

The RMSD plots of native PanK Cα-atoms without a bound ligand were constant between 1.25 Å and 2.00 Å (Fig. 3B). The RMSF of the unbound PanK residues was below 2.5 Å (Fig. 3B). A comparison of the RMSDs of the PanK-norajmaline complex and that of the unbound native PanK indicated that the binding of the ligand results in changes in slight protein confirmation. Likewise, the RMSF of the two systems showed that smaller fluctuations were observed on PanK C-α-residues that interacted with the ligand’s atoms (Fig. 2B, and Fig. 3B).

**Fig. 4:**
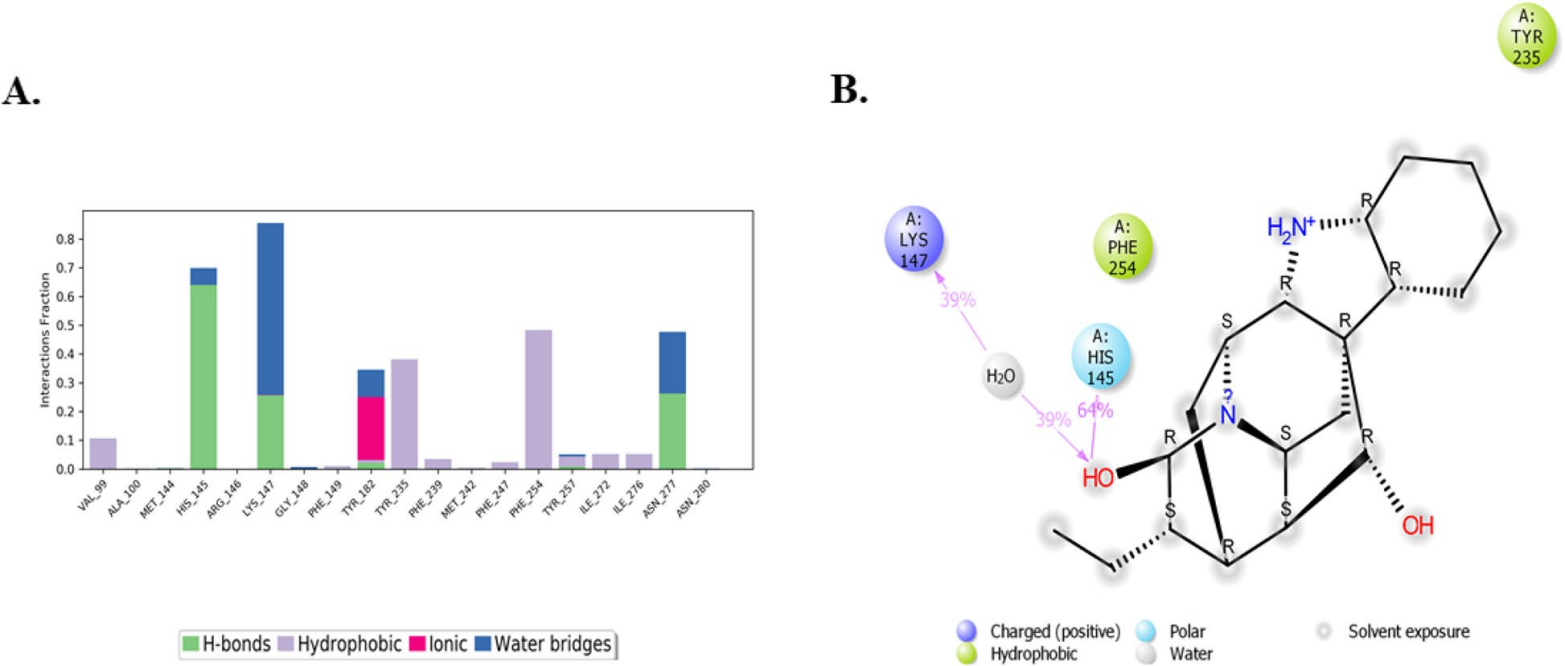
In **A**, the interaction fraction of the PanK residues with norajmaline is displayed. In **B**, the non-covalent interactions between the ligand and the PanK residues that occurred for more than 30 ns of MD simulation duration are displayed

The interaction of PanK-norajmaline was mainly due to hydrogen bonds (His145, Tyr235, and Asn277), hydrophobic contacts (Val99, Try235, Phe239, Met242, Phe247, Phe254, Try257, Ile272, Ile276), ionic interactions (Tyr182) and water bridges (His145, Lys174, Try182, Try257, and Asn277) (Fig. 4A). The contacts which occurred for more than 30% of the simulation time are charged (occurrence = 39%), polar interactions (occurrence = 64%), and hydrophobic interactions were also predominant since the ligand was in a hydrophobic pocket of PanK as depicted in Fig. 4B.

### Post-MD simulations MM-GBSA (molecular mechanics generalized born surface area) binding energy calculations

The post-MD simulations binding free energy (ΔG_bind_) of the PanK-norajmaline complex were evaluated using the MM-GBSA method. The PanK-norajmaline complex exhibited an MM-GBSA dgBind energy of –58.73 kcal/mol (Table 6), while the control ligand exhibited –67.70 kcal/mol (Table 6). The MM-GBSA supports the stability of the complex as shown in RMSD plots (Fig. 2).

**Table 6:** MM-GBSA (molecular mechanics generalized born surface area) ΔG_bind_ calculations

**Compound ID**	**MM-GBSA (kcal/mol)** ΔG_bind_
Control ligand (1f)	–67.70
Norajmaline	–58.73

### Cytotoxicity effects of crude plant extracts against MDA-MB 231 cells

**Fig. 5:**
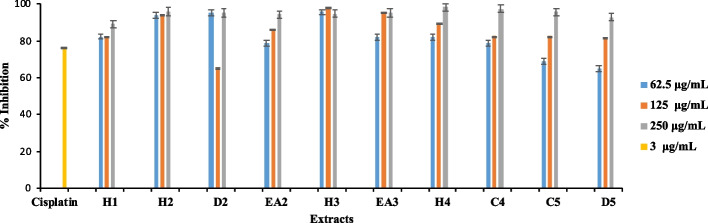
Cytotoxicity activity of R. caffra (H2, D2 and EA2), S. molle (H3 and EA3), Z. mucronata (H4 and C4), and S. petersiana (C5 and D5) (62.5, 125 and 250 µg/mL) and cisplatin (3 µg/mL) as a control drug against MDA-MB 231 triple-negative breast cancer. Results represent the mean ± Standard deviation of triplicate determinations

The crude extracts from medicinal plants, *S. brachypetala, R. caffra, S. molle, Z. mucronata,* and *S. petersiana* (62.5, 125 and 250 µg/mL) were studied for their anticancer effects on MDA-MB-321 cells, as shown in Fig. 1. Some of the intermediate polarity extracts from *R. caffra* (H2, D2 and EA2), *S. molle* (H3 and EA3), *Z. mucronata* (H4 and C4), and *S. petersiana* (C5 and D5) showed over 50% inhibition at the lowest concentration (62.5 µg/mL). *R. caffra* (H2, EA2) and *S. molle* (H3) extracts showed inhibition of over 80% at all concentrations. All the medicinal plant crude extracts showed they could slow the growth of MDA-MB-231 triple-negative breast cancer cells. The extracts that were effective (as shown in Fig. 5) were studied further to find the lowest concentration where they could inhibit 50% of growth.

### Cytotoxic effects of plant extracts against hepg2/C3A liver and vero monkey kidney cell lines

**Fig. 6:**
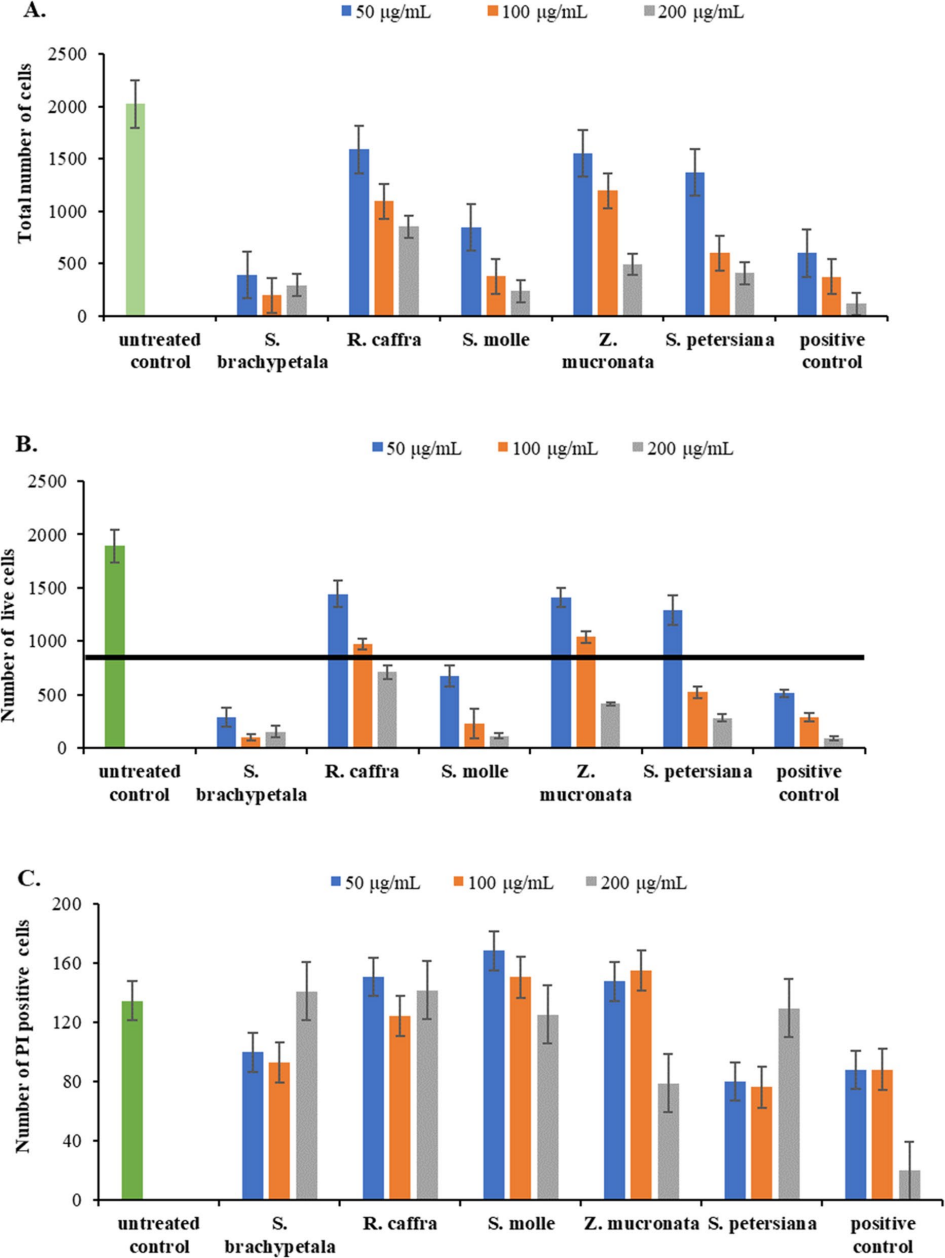
Cytotoxicity of 5 extracts and Melphalan (10, 20, and 40 μM) as the reference drug against HepG2A/C3A after 48 h of exposure. Results displayed as total number of cells (**A**), number of cells stained with Hoechst 33342 only (**B**), and Hoechst 33342 and PI (**C**)

**Fig. 7:**
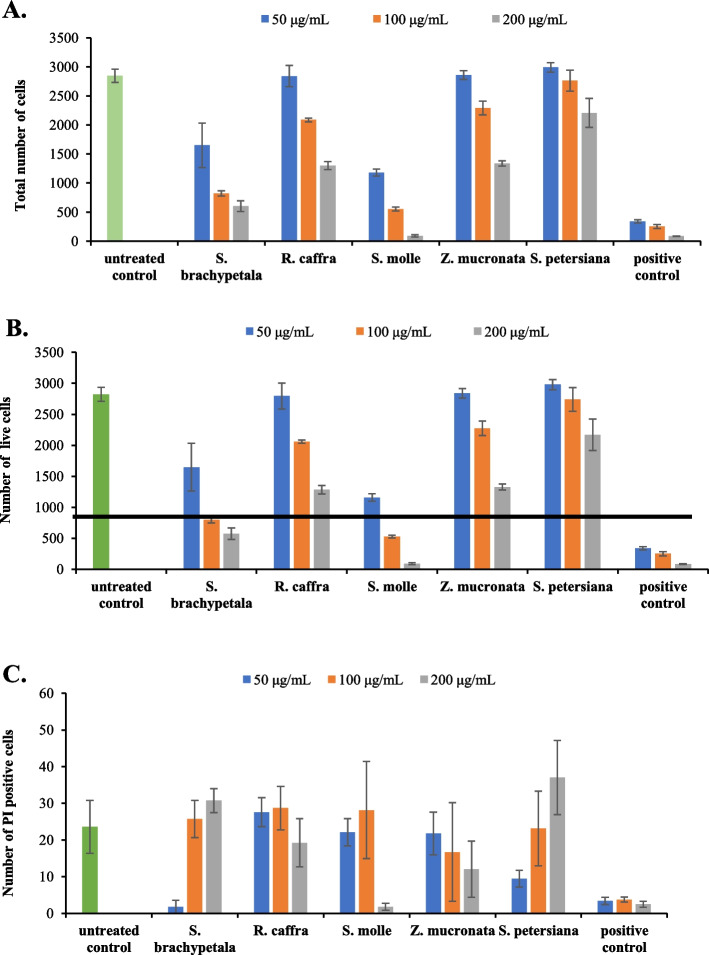
Cytotoxicity of 5 extracts and Melphalan (10, 20, and 40 μM) as the reference drug against Vero cells after 48 h of exposure. Results displayed as total number of cells (**A**), number of cells stained with Hoechst 33342 only (**B**), and Hoechst 33342 and PI (**C**)

The crude extracts of each plant species were tested for potential cytotoxicity against HepG2/C3A and Vero cell lines. The distinction between these cell lines lies in the fact that HepG2/C3A represents a liver cell model, while Vero serves as a model for normal monkey kidney cells. In this study, the cytotoxicity test measured the number of live cells after treatment of the two cell lines with the plant extracts. The black horizontal lines in Fig. 6B and 7B indicate half (50%) of the untreated control cells and extracts exhibiting cytotoxic potential are shown in Fig. 6B and 7B as having viable (live) cell numbers below the black line.

Figure 6 shows that *R. caffra* is the least toxic, followed by *Z, mucronata*, *S. petersiana*, *S. molle* and *S. brachypetala*, the most toxic against HepG2/C3A cells at 50 and 200 µg/mL. Cytotoxicity against Vero cells was also determined, and the results depict that all plant crude extracts appear to be less toxic against the normal cell line (Vero cell) at the lowest concentration (50 µg/mL) tested and *S. petersiana* exhibited no harmful effect at all concentrations tested (50–200 µg/mL). However, *S. brachypetala* and *S. molle* were toxic to the Vero cells at a higher concentration (100 and 200 µg/mL) (Fig. 7). All crude plant extracts that showed lower cytotoxicity against the HepG2/C3A and the Vero cell lines were further investigated by determining their IC_50_ values on MDA-MB 231 triple-negative breast cancer.

### Anti-proliferation activity of selected plant extracts against MDA-MB 231 cell line

**Fig. 8:**
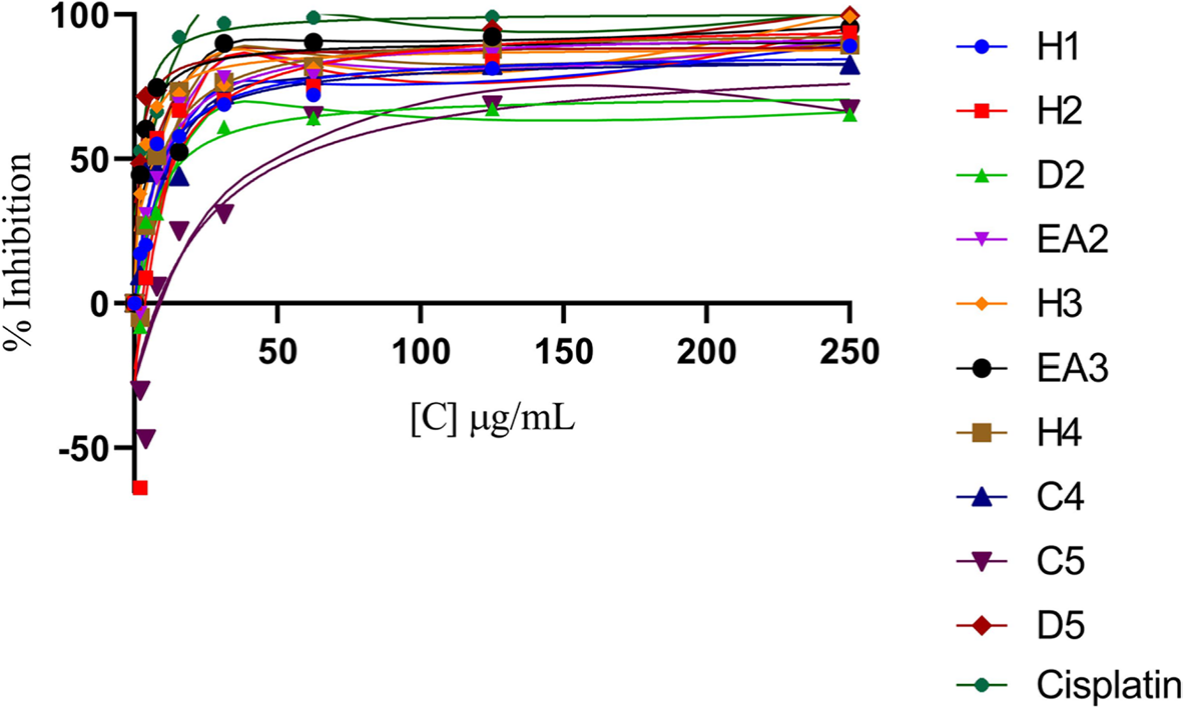
Dose–response curve of the cytotoxicity of the medicinal plants S. brachypetala (H1), R. caffra (H2, D2, EA2), S. molle (H3, EA3,), Z. mucronata (H4, C4), S. petersiana (C5, D5), and the reference control drug (Cisplatin) against MDA-MB 231 triple-negative breast cancer cell line. Cells were treated with incubated varying concentrations of the selected crude extract for 48 h, after which an MTT assay was performed. The data points shown represent the mean ± the standard deviation of technical and biological triplicate repeats. The data were analysed using GraphPad Prism 8 software to obtain the IC50 concentrations

Active crude plant extracts against MDA-MB 231 were chosen to be evaluated further to determine their 50% inhibitory concentration (IC_50_). Cisplatin was used as the reference drug and the concentrations at which the crude plant extracts were tested are as follows: 3.906, 7.8125, 15.625, 31.25, 62.5, 125 and 250 µg/mL. The chloroform crude extract of *S. petersiana* (C5) showed the highest IC_50_ of 26.26 ± 2.325 µg/mL, followed by the hexane crude extract of *R. caffra* (H2) at 8.625 ± 0.163 µg/mL. However, the dichloromethane crude extract of *S. petersiana* (D5) showed the lowest IC_50_, 1.525 ± 0.458 µg/mL, even lower than the reference drug, cisplatin (2.017 ± 0.09 µg/mL) (Fig. 8 and Table 7).

**Table 7:** Summary of IC_50_ values of the cytotoxic effect of cisplatin and H1, H2, D2, EA2, H3, EA3, H4, C4, C5 and D5 crude extracts against MDA-MB 231 triple negative cancer cell line

Sample	Extract code*	IC_50_ (µg/mL)	*p* value
*S. brachypetala*	**H1**	7.425 ± 0.911	0.0138
*R. caffra*	**H2**	8.625 ± 0.370	0.0068
*R. caffra*	**D2**	6.829 ± 0.370	0.0203
*R. caffra*	**EA2**	7.617 ± 0.755	0.0266
*S. molle*	**H3**	2.646 ± 0.725	0.6516
*S. molle*	**EA3**	2. 426 ± 0.141	0.7672
*Z. mucronata*	**H4**	7.019 ± 1.210	0.0375
*Z. mucronata*	**C4**	7.227 ± 0.213	0.0157
*S. petersiana*	**C5**	26.26 ± 2.325	0.0157
*S. petersiana*	**D5**	1.525 ± 0.458	0.7225
**Cisplatin**	**-**	2.017 ± 0.090	0.0044

^*^Extract code: *H*-Hexane, *D* Dichloromethane, *EA* Ethyl acetate, *C* Chloroform

### Determination of mode of cell death using annexin-V and PI staining

**Fig. 9:**
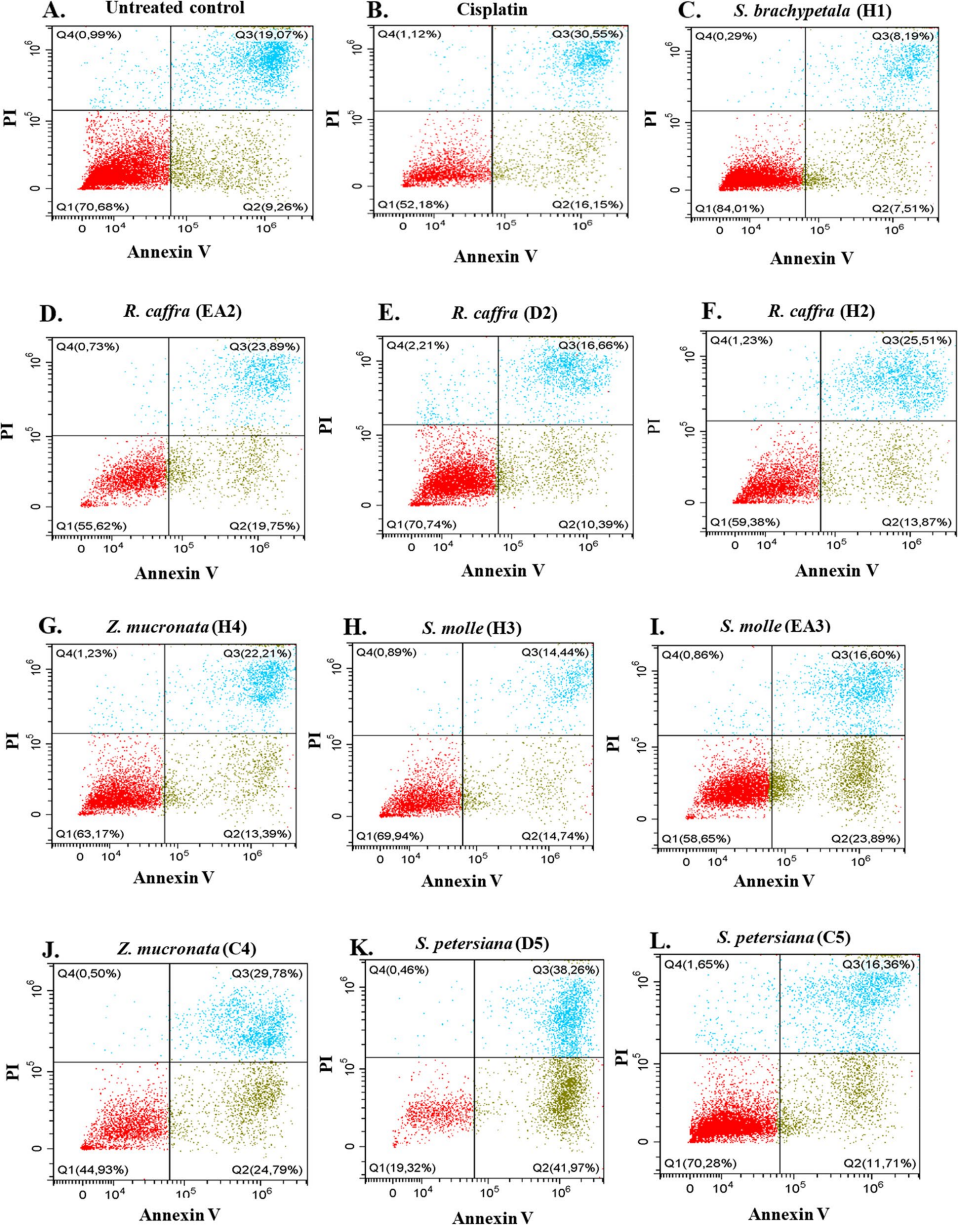
**A**-**F** Effect of crude extracts from S. brachypetala (H1), and R. caffra (H2, D2, EA2) compared to untreated control and cisplatin (positive control), on stained MDA-MB 231 to identify mode of cell death using flow cytometry. **G**-**L** Effect of crude extracts from S. molle (H3, EA3), Z. mucronata (H4, C4), and S. petersiana (C5, D5) compared to untreated control and cisplatin (positive control), on stained MDA-MB 231 cells to identify mode of cell death using flow cytometry

**Fig. 10:**
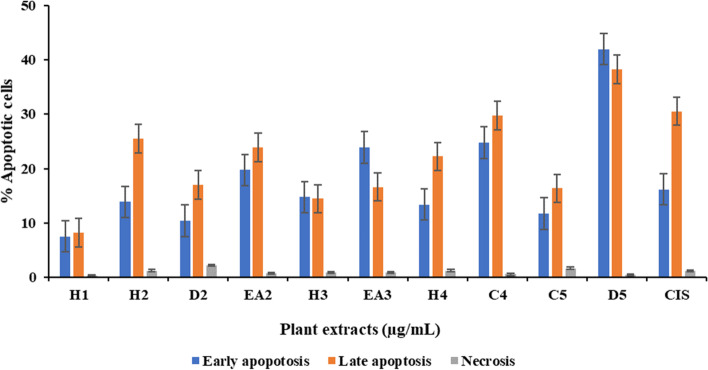
Percentage of MDA-MB cells at early, late apoptotic and necrosis mode of death after being treated with S. brachypetala (H1), R. caffra (H2, D2, EA2), S. molle (H3, EA3), Z. mucronata (H4, C4), S. petersiana (C5, D5), ad Cisplatin (CIS) on stained MDA-MB 231 cells

The plant crude extracts that showed growth inhibitory activity against MDA-MB 231 triple-negative breast cancer cell line were further investigated to determine the mode of cell death, using the Annexin V and propidium iodide (PI) kit (Invitrogen, Thermo Fisher Scientific). Annexin V binds to phosphatidylserine (PS), which translocated from the inner cell membrane to the outer cell membrane during early apoptosis. Propidium iodide enters dead cells via their compromised cell membranes and stains the nucleus of dead cells [31]. Flow cytometry was used to analyze the mode of cell death (Figs. 9 and 10).

The untreated control showed a high background apoptosis of 9.26% apoptosis. Cisplatin (control drug) induced 16.15% and 1.12% early apoptosis and necrosis, respectively (Fig. 9A), while the untreated control showed 9.26% and 0.99% early apoptosis and necrosis, respectively (Fig. 9A). The plant crude extracts of *R. caffra* (EA2, 19.75%), *S. molle* (EA3, 23.89%), *Z. mucronata* (C4, 24.76%) and *S. petersiana* (D5, 41.97%) induced a higher percentage early apoptosis than cisplatin. D5 showed the best activity against the MDA-MB 231 cancer cells, with 41.97% early apoptosis. All extracts induced less than 2.5% necrosis under the condition screened (Figs. 9 and 10).

## Discussion

Plants efficiently deploy sophisticated defense mechanisms to fight infections, which renders them immune to numerous pathogenic microorganisms. Phytocompounds are among the arsenal used by plants for combating microbial infections. This study explored the phytocompounds present in crude extracts from *S. brachypetala, S. petersiana, Z. mucronate*, *R. caffra,* and *S. molle* to decipher their antimycobacterial activity. The crude extracts from *R. caffra* exhibited strong growth inhibitory activity against *M. aurum* A + (MIC = 0.02–0.5 mg/mL) (Table 2). Furthermore, *R. caffra* dichloromethane extracts exhibited potent growth inhibition against *M. tuberculosis* H37Rv (MIC = 0.25–0.125 mg/mL) (Table 3). Traditional practitioners in Limpopo use *R. caffra* concoctions to treat a wide range of diseases. Findings in this study confirm that extracts from *R. caffra* strongly inhibit *M. tuberculosis* as previously reported [10]. In this study, the chemical constituents from *R. caffra* extracts that might have synergistically contributed to the potent antimycobacterial activity were tentatively identified. Results from this study confirm the literature that showed that *R. caffra* is rich in alkaloids (Table 4) [32]. Our study also supports a report by Tlhapi et al., [33] which identified and isolated raucaffricine from *R. caffra* extracts. Extracts constituting alkaloids have previously been investigated for pharmacological activities and have been discovered to exhibit potent antimicrobial efficacy [10, 34].

In this study, crude extracts from *S. molle* presented strong antimycobacterial efficacy, resulting in high susceptibility patterns with low MIC values ranging from 0.02–0.5 mg/mL for *M. aurum* A + and 0.25–0.125 mg/mL for *M. tuberculosis* H37Rv) shown in Tables 2 and 3. A study by Turchette et al., [35] demonstrated the inhibitory activity of extracts from *S. molle* against gram-positive bacteria (*Bacillus subtilis*). Another report by Bernardes et al. [36], demonstrated the significant susceptibility of *Mycobacterium bovis* BCG to the methanolic extract of *S. terebinthifolius* which belongs to the *Schinus* genus*.* To the best of our knowledge, this study is the first to report *in-vitro* anti-*M. tuberculosis* H37Rv activity of *S. molle.* In this study, tentative identification of phytocompounds constituting *S. molle* extracts showed the presence of sesquiterpene, triterpene, terpene, triterpenoid, and triterpenoid saponin. Our study agrees with the phytochemical profiling of *S. molle* by various studies which showed the presence of a wide array of potentially bioactive compounds from classes of compounds, including sesquiterpenes, terpenes, and triterpenes [12, 13, 35]. Tannins, flavonoids, steroids, and catechins have also been reported to contribute to the antimicrobial activity of the *Schinus* genus [37]. Interestingly, oleanolic acid, a triterpenoid tentatively identified in this study (Table 8) was shown in other reports to exhibit anti-*M. tuberculosis* and reduce hepatotoxicity [38–41]. In addition, sesquiterpenes, terpenes, triterpenes, and triterpenoids that were also tentatively identified are well characterized for antimicrobial activity and may have contributed to the anti-*M. tuberculosis* activity.

**Table 8:** Tentatively identified compounds present in *S. molle* crude extract

RT (min)	Peak height	Precursor *m/z*	Molecular formula	Error ppm	Compound	Class
5.4580	4281.99	237.1853	C_15_H_24_O_2_	1.6611	Aubergenone	Sesquiterpene
6.1084	3686.87	203.1800	C_15_H_22_	2.8201	Beta-Spathulene	Sesquiterpene
10.6350	6452.70	471.3477	C_30_H_46_O_4_	1.7269	Semialatic acid	Triterpene
9.3080	3843.11	205.1953	C_15_H_24_	1.0867	Beta-Caryophyllene	Terpene
9.9397	11675.21	453.3369	C_30_H_44_O_3_	1.2749	Pistacigerrimone	Triterpenoid
11.6107	1499.63	455.3534	C_30_H_46_O_3_	3.1360	Isomasticadienonic acid	Triterpenoid
12.0929	1943.07	457.3684	C_30_H_48_O_3_	1.7010	Oleanolic acid	Triterpenoid saponin
13.2405	1172.57	441.3720	C_30_H_48_O_2_	–1.6018	28-Hydroxy-beta-Amyrone	Triterpenoid

The dichloromethane and methanol extracts *of Z. mucronata* showed strong activity against *M. aurum* A + , with MIC values of 0.04 mg/mL and 0.25 mg/mL, respectively. However, poor efficacy was observed for *Z. mucronate*, *S. brachypetala,* and *S. petersiana* against *M. tuberculosis* H37Rv. Results in our study agree with a report by Mativandlela et al. [42], which illustrated that bark extracts of *Z. mucronata* exhibit poor activity against *M. smegmatis*. On the contrary, other reports demonstrated that a combination of the *Z. mucronate* leaf, bark, and root extracts exhibit potent activity (MIC ≤ 1 mg/mL) against *M. tuberculosis* [2, 43]*.* The poor activity observed against *M. tuberculosis* by crude extracts from *Z. mucronate*, *S. brachypetala,* and *S. petersiana*, does not completely reflect on lack of activity of the extracts in vivo because some of the compounds may be enzymatically activated or transformed in vivo*.* The transformed intermediates may then be potent against *M. tuberculosis*.

Results from the rigorous VSW returned only one hit compound (norajmaline) (Table 5). In addition, norajmaline did not violate the *Rule of Five*. Lipinski’s Rule of five defines the potential drug-likeness of a compound based on the relationship between physiochemical and pharmacokinetics parameters active [44]. The ADME properties of norajmaline observed in this study (Table 5) were moderate but within acceptable ranges [45]. There is a high failure of drugs in the clinical phases because of poor pharmacokinetic properties [46, 47]. Thus, this investigation used the ADME parameters as filters to avoid the identification of false hits (Table 5). The molecular docking regimes with increasing precision in this study were performed as a further refining stage, with the objective of obtaining a hit molecule(s) based on extra-precision scores and gaining molecular insight into the binding mechanism. The XP docking score of norajmaline was –7.47 kcal/mol (Table 5). Computational screening provides an efficient approach to identifying, characterizing, and modifying potential drug leads [26, 48].

To expand the essential knowledge about the binding dynamic trends of the phytocompound against *M. tuberculosis* PanK, molecular dynamics simulations, and ΔG_bind_ were computed. Norajmaline formed a relatively stable complex with PanK with RMSD below 3 Å (Fig. 2). A comparison of the unbound native PanK RMSD and that of PanK-norajmaline revealed that the interaction of norajmaline with the residues of the binding pocket of PanK resulted in a slight structural change of PanK (Fig. 2, and Fig. 3). Norajmaline exhibited high affinity (ΔG_bind_ –58.73 kcal/mol) to the hydrophobic binding domain of PanK based on the MM-GBSA calculations (Table 6). The control ligand used in this study **1f** is an engineered triazole competitive inhibitor of PanK [23]. A comparison of the MD simulations of the 1f and norajmaline revealed that the 1f had a higher affinity (ΔG_bind_ –67.70 kcal/mol) to the PanK binding pocket than the norajmaline (ΔG_bind_ –58.73 kcal/mol) as shown by the post-MM-GBSA (Table 6). For a natural product, norajmaline has an interestingly high affinity. The pre-MM-GBSA ΔG_bind_ of the norajmaline-PanK complex (–37.64 kcal/mol) is more than that of post-MM-GBSA ΔG_bind_ –58.73 kcal/mol. Molecular knowledge derived from virtual screening of phytochemicals revealed that norajmaline may potentially competitively inhibit *M. tuberculosis* PanK. On the other hand, norajmaline can be further modified to enhance the affinity and ADME properties while at the same time enhancing activity.

The current study investigated the cytotoxicity properties evaluated by *S. brachypetala, R. caffra, S. molle*, *Z. macronata*, and *S. petersian* indigenous to Limpopo Province, South Africa against MDA-MB 231 triple-negative breast cancer cell line. The hexane crude extracts of *S. brachypetala* (H1), *R. caffra* (H2, D2 and EA2), *S. molle* (H3 and EA3), *Z. macronata* (H4 and C4), and *S. petersian* (C5 and D5) induced > 50% cell growth inhibition against the MDA-MB 231 triple-negative breast cancer line at 62.5, 125 and 250 µg/mL. All the plant extracts had an IC_50_ value less than 30 µg/mL, which meets the criteria set by the American National Cancer Institute for a potent extract [49, 50]. According to these guidelines, an IC_50_ value of less than 30 µg/mL indicates that the extract has the potential to effectively halt the growth of the cancer cells [49, 50].

Toxicological assays on medicinal plants of this study indicate that crude extracts exhibited low toxicity towards Vero monkey kidney cells, which agreed with previous findings of Tlphapi et al., [11], where they found that the *R. caffra* crude extract, fractions and pure compounds did not display any cytotoxic effects at a concentration of 50 μg/mL against HeLa cells. This is also supported by the observation from Sigidi et al., [51] where *S. petersiana* had the lowest toxicity against Vero monkey kidney cells at a concentration lower than 50 µg/mL. It showed that these plant species could be further experimented with since they do not display toxicity against healthy cells. In comparison, a previous study found that *Z. mucronata* extracts had low toxicity with IC_50_ values ranging from 150 to 250 mg/mL against Vero monkey kidney and MeWo cells [51]. Moreover, an aqueous extract of *Z. mucronata* was found to have lower toxicity against HepG2/C3A cells with an IC_50_ value greater than 100 μg/mL [52]. These findings reinforce the observed low toxicity of *Z. mucronata* on HepG2/C3A cells in this study (Figs. 6 and 7).

However, the hexane extract of *S. molle* showed the highest cytotoxic inhibition activity on the HepG2/C3A cell as reported by Nagah et al*.*, (2021) [53] which is consistent with the results obtained in this study (Fig. 6). The findings of the current study were supported by the study of Dzoyem et al., [43], which found that *Z. mucronata* and other indigenous plants were relatively safe compared to the positive control when tested on Vero cells. Additionally, bark extracts from *S. brachypetala* and *Z. mucronata* were found to be nontoxic against brine shrimp [43], which concurs with the results observed in this study on Vero cells (Fig. 7). Contrarily, Ruffa et al., [54] reported that the methanolic extract of *S. molle* was highly lethal to the human hepatoma HepG2 cell line, in contrast to extracts from other medicinal plants. This observation was supported by Hailan et al., [55], where nanoparticles synthesized from *S. molle* extracts were found to be highly toxic against HepG2 cells [56]. In that study, they attributed the high cytotoxicity of *S. molle* to several terpenoid compounds identified in its essential oil [55]. Due to their toxic properties, *S. molle* extracts are commonly used as insecticides [57, 58].

The results of this study indicate that various solvent extracts from five different plants (*S. brachypeta, R. caffra, S. molle, Z. mucronata*, and *S. petersiana*) have varying levels of cytotoxic activity against cancer cells. The IC_50_ values of these extracts ranged from 1.525 ± 0.458 to 26.26 ± 2.325, with the dichloromethane extract of *S. petersiana* (D5) and the ethyl acetate extract of *S. molle* (EA3) showing the highest potency with IC_50_ values of 1.525 ± 0.458 and 2.426 ± 0.141, respectively. Other extracts showed moderate antiproliferative activity with IC_50_ values ranging from 6.829 ± 0.37 to 8.625 ± 0.37. The control drug, cisplatin, showed potent antiproliferative activity with an IC_50_ value of 2.017 ± 0.09. It is worth noticing that the dichloromethane extract of *S. petersiana* (D5) and the ethyl acetate extract of *S. molle* (EA3) (2.426 ± 0.141) showed similar IC_50_ as the control drug (Fig. 8 and Table 7).

The results of this study (Fig. 8 and Table 7) are consistent with previous findings in the literature, which have shown that various plant extracts have antiproliferative activity against cancer cells. Nguefack et al., [59] found that extracts from the root bark of *Rauvolfia vomitoria*, a close relative of *R. caffra*, showed significant antiproliferative activity against human breast cancer cells (MCF-7) [59]. Another study by Agbo et al., found that the ethanol extract of *Ziziphus mauritiana*, a close relative of *Z. mucronata*, showed antiproliferative activity against human liver cancer cells (HeLa cells) and breast cancer cells (MCF-7 cells) [60]. The IC_50_ values of the plant extracts in the present study are also similar to those reported for other plant-based anti-cancer agents. A study by Kim et al., [61] found that the IC_50_ value of an ethanol extract from *Scutellaria baicalensis*, a traditional medicinal plant, was 2.55 ± 0.35 against human breast cancer [61]. Another study by Kim et al., found that the IC_50_ value of a methanol extract from *Tripterygium wilfordii*, another traditional [62] medicinal plant, was 4.66 ± 0.54 against human leukaemia cells (HL-60 cells) through the mitochondrial pathway [62]. The results of this study indicate that various solvent extracts from *S. brachypeta, R. caffra*, *S. molle*, *Z. mucronata*, and *S. petersiana* exhibit varying degrees of antiproliferative activity against the MDA-MB 231 triple-negative cancer cells. To the best of our knowledge, this study is the first to report the cytotoxicity activities of these five medicinal plants from Limpopo against MDA-MB 231 triple-negative breast cancer. This highlights the potential of these plant extracts as alternative sources of anti-cancer agents against triple-negative breast cancer, but further studies are needed to confirm their activity and determine their potential as therapeutic agents [28].

Apoptosis is a type of cell death linked to cancer [63, 64] Apoptosis signalling has been used as a cancer therapy [63–66] The current study evaluated the apoptotic effects of various plant species on cancer cells using Annexin V and propidium iodide staining procedure via flow cytometry. The results were presented in contour plots for apoptotic and necrotic cell death (Figs. 9 and 10). Cisplatin (16.15% apoptosis and 1.12% necrosis) was used as a positive control and 0.25% DMSO as an untreated control. The study found that *S. petersiana* extract (D5) exhibited an early apoptotic stage at 41.97% (Figs. 9 and 10), which is in agreement with literature that revealed the anticancer potential of the extracts [67]. *S. brachypetala* (H1) was observed to have a high percentage of live cells (84%) with low apoptotic and necrotic activities (7.51% and 0.29%, respectively) (Figs. 9C and 10) compared to other extracts, although phenolic compounds from *S. brachypetala* have been reported to have therapeutic effects against Alzheimer’s disease [68, 69] The essential oil from *R. caffra* was found to be effective against MCF-7 breast cancer cells, possibly due to its high antioxidant activity [70, 71]. *S. molle* extracts were reported to have anticancer effects on human leukaemia cells (HL-60 cells) [12] and showed toxicity to the U-937 cell line and anti-tumour activity against human leukaemia monocyte lymphoma [71]. Silver nanoparticles synthesized from *S. molle* extracts were also observed to exhibit potential anticancer activity against HepG2 cells [55]. All these findings agree with the findings from this current study. It is worth noting that the chloroform extracts from *Z. mucronata* (C4) were observed to induce higher late apoptotic cell death (29.79%) than the hexane extracts (H4) (22.21%) (Figs. 9J, 9G and 10), suggesting that compounds with anticancer activity could have intermediate or opposite polarity [72]. It is worth noting that the findings from this current study revealed that four plant extracts induced higher levels of apoptosis compared to the control drug, cisplatin. The highest level of apoptosis was observed with the dichloromethane extract of *S. petersiana* (D5) at 41.97%, followed by the chloroform extract of *Z. mucronata* (C4) at 24.74%, the ethyl acetates crude plant extracts of *S. molle* (EA3) at 23.89%, and *R. caffra* (EA2) at 19.75%. The control drug, cisplatin, induced apoptosis at 16.15%. These findings suggest that the crude plant extracts tested in this study could potentially be used as alternative treatments for triple-negative breast cancer.

## Conclusion

Medicinal plants are an invaluable source of potent bioactive anti-mycobacterial phytocompounds. *R. caffra* and *S. molle* which are prescribed by traditional healers in Limpopo are potent against *M. tuberculosis* and assist the developing community in alleviating and treating TB. The integration of targeted virtual screening can rapidly and effectively be employed to identify potential lead compounds. Rigorous virtual screening comprising many filtering parameters reduces the chances of obtaining false positives. Norajmaline showed exceptionally high affinity to PanK and may be further modified to enhance affinity and ADME properties. This study offered a glimpse into the cytotoxic activities and antioxidant attributes of five selected medicinal plants from Limpopo, South Africa, against MDA-MB triple-negative breast cancer. The findings indicated that apoptosis, was the form of cell death against MDA-MB triple-negative breast cancer.

### Recommendation

The crude extracts of the plants comprise a wide variety of compounds that can further be separated and experimentally evaluated for anti-mycobacterial activity. Although the findings indicate that the selected medicinal crude plant extracts have the potential to treat triple-negative breast cancer, more exploration is needed to delve into the plants' mechanisms of action and isolate the bioactive elements responsible for the plant's anticancer properties in the in vitro study.

## Supplementary Information

**Additional file 1: Appendix.**

## Data Availability

The datasets used, and analyses during the current study are available from the corresponding authors upon reasonable request.

## Abbreviations

MM-GBSA: Molecular mechanics generalized born surface area
RMSD: Root mean square deviation
ADME: Absorption, distribution, metabolism, and excretion
RMSF: Root mean square fluctuation
LC-QTOF-MS/MS: Liquid chromatography tandem quadrupole time-of-flight mass spectrometry
OPLS4: Optimized potentials for liquid simulations 4
NPT: Isothermal isobaric ensemble
TIP3P: Transferable intermolecular potential 3P
