# Supplementary material for: In-silico and in-vitro assessments of some fabaceae, rhamnaceae, apocynaceae, and anacardiaceae species against Mycobacterium tuberculosis H37Rv and triple-negative breast cancer cells
Source: BMC Complement Med Ther. 2023 Jul 1;23:219. doi: 10.1186/s12906-023-04041-5 (PMC10314437; doi:10.1186/s12906-023-04041-5)
Supplement: Supplementary file 1 — Additional file 1: Appendix. [file 12906_2023_4041_MOESM1_ESM.docx]

**APPENDIX**

**Table A1.** Botanical name and voucher specimens of medicinal plants that used in this study.

| **Voucher specimen no.** | **Plant name** | **Plant parts** | **Solvent** | **Extract** |
| --- | --- | --- | --- | --- |
| MMV004 | *Schotia brachypetala Sond* | Roots | Hexane | H1 |
| MMV003 | *Rauvolfia caffra Sond* |  | Hexane | H2 |
|  |  | Barks | Dichloromethane | D2 |
|  |  |  | Ethyl acetate | EA2 |
| MMV005 | *Schinus molle Linnaeus* |  | Hexane | H3 |
|  |  | Leaves | Ethyl acetate | EA3 |
| MMV002 | *Ziziphus mucronata Willd* |  | Hexane | H4 |
|  |  | Leaves | Chloroform | C4 |
| MMV001 | *Senna petersiana (Bolle) Lock* |  | Chloroform | C5 |
|  |  | Leaves | Dichloromethane | D5 |
